# Case of Polypus Protruded at the Time of Labor

**Published:** 1812-06

**Authors:** Thomas Denman

**Affiliations:** Mount-street


*For the Medical and Physical Journal.*

CASE *of* POLYPUS *protruded at the* TIME *of* LABOR.

ON the 17th of March, 1811, the wife of ———, the mother of many children, was delivered of a living female child, after an unusually severe labor of several hours continuance. During the labor, a tumor was pushed down

 before before the head of the child, and toward the *ossa pubis.* The same kind of tumor, though far less in bulk, had been observed in the two immediately preceding labors; but, as that had been retracted after the birth of the children, and was followed by no inconvenience, particular attention was not paid to it. On the present occasion the tumor was not retracted, but remained without the *os externum.* On examination, it was found to be a soft fleshy substance, of a deep red color, near the size of half the head of a new-born infant, and it seemed to have been bruised by the pressure it had undergone in the passage of the child through the pelvis, being partly covered with a thick layer of coagulated blood. As the circumstance was new to two practitioners who attended the patient, a third of much professional eminence was called in on the evening of the same day, who entertained no doubt of its being a *polypus.*

On the following morning a ligature, drawn moderately tight, was applied for the purpose of promoting its separation. For a few hours after the application of the ligature, the patient continued easy, but towards evening she became restless, and so continued through the ensuing night. On the 19th, the tumor being apparently little altered, and the ligature slackened, another was applied, with the view of more speedily and effectually stopping all circulation through it. But so much uneasiness was occasioned by the new ligature, that it was judged expedient to remove it in the evening, and she was instantly relieved from the pain she had suffered. To quiet the symptoms of irritation which from the beginning had been considerable, from ten to twenty drops of tinctura opii had been given every six hours; and, throughout her illness, the bowels were occasionally relieved by clysters. The tumor was now so much increased in size as to render the introduction of the catheter, which from the time of her delivery had been necessary, very difficult; and the surface of it had assumed a darker appearance, particularly toward the back part of it.

From this time the symptoms of debility and irritation were much increased; her pulse was feeble, irregular, and not less than 120 in a minute; the skin hot, but yet always dry; and her thirst was urgent. On the 23d, in the morning, the patient was attacked with convulsions, which returned at short intervals during the whole day, and for many hours of the following night. While these continued, she was able to take very little nourishment, and scarcely to speak intelligibly. Early in the morning of the 24th the convulsions left her, and she began to revive: her pulse was more strong and regular, and the power of taking nourishment, ment, and of speaking more distinctly, returned. She had, indeed, a craving for food, but, when this was incautiously given, her stomach was much incommoded by it.

By a profuse, fœtid, and somewhat purulent, discharge, the tumor was soon considerably lessened. In a few days the anterior part of it sloughed superficially, and the whole of it had a fresher appearance. Still it occasioned much inconvenience, and there was a frequent occurrence of forcing pains, as if the *uterus* was in action to exclude something more out of its cavity, or to eject the tumor from the vagina.

In this state the patient continued for several weeks, suffering now and then much pain and distress, unable from weakness to assist herself in almost any degree, and the bulk of the tumor was nearly stationary. We were deterred from applying another ligature by the aggravated disturbance and irritation which had been raised by the former ones. But, about nine weeks after her delivery, we took what was considered as a favorable opportunity, and ventured to apply another ligature. This produced much the same symptoms as the former ones; much local and constitutional irritation, a sloughing of the surface of the tumor, with increased fœtid discharges; but there was no material diminution of the size of the tumor, and this ligature was therefore removed.

We were now hopeless, at least very doubtful, of the possibility of a perfect cure, and for the present satisfied ourselves with endeavoring to alleviate the sufferings, and support the strength, of the patient. In these respects we were ably assisted by the constant attention and care of affectionate relatives and attendants.

About four months after her delivery, the general health of the patient being much improved, she herself was very desirous that another attempt should be made to extirpate the tumor, which was very troublesome, and on many accounts extremely disagreeable. Accordingly another ligature of strong packthread was fixed and drawn very tight round the neck of the tumor, now much smaller, and more easily accessible, than when the former attempts were made. The pain, which from the first protrusion of the tumor had been in a greater or less degree almost incessant, was immediately abated, and after a few days the bulk of it sloughed away. But a small portion of it, exterior to the ligature, had a fresh appearance, and looked as if it were disposed to sprout again. To this another ligature was applied, which, after a few days more, completely removed every part of the tumor. From that time the patient was gradually though slowly restored to perfect health, and resumed her wonted occupations.

She

She has since menstruated regularly, and there remains not the least appearance or symptom of disease, except that she has sometimes a slight sense of fulness in the umbilical region.

*Observation.* It does not perfectly accord with the wish of Dr. Pope, and Mr. Tothill, surgeon at Staines, that their names should be mentioned on this occasion; but their management of the case was so intelligent and judicious, that it would not be just to conceal them. Among several other things worthy of notice, one obvious, plain, and useful, lesson is to be learned—that, in a similar case, it would be highly improper to pass a ligature round the stem of the *polypus,* before the local and constitutional irritability was appeased.

*Mount-street,* THOMAS DENMAN, M.D.

*April 29th,* 1812.

